# Smaller, softer, lower-impedance electrodes for human neuroprosthesis: a pragmatic approach

**DOI:** 10.3389/fneng.2014.00008

**Published:** 2014-04-16

**Authors:** Elisa Castagnola, Alberto Ansaldo, Emma Maggiolini, Tamara Ius, Miran Skrap, Davide Ricci, Luciano Fadiga

**Affiliations:** ^1^Robotics, Brain and Cognitive Sciences Department, Istituto Italiano di TecnologiaGenoa, Italy; ^2^Struttura Complessa di Neurochirurgia, Azienda Ospedaliero-Universitaria Santa Maria della MisericordiaUdine, Italy; ^3^Section of Human Physiology, Department of Biomedical Sciences, University of FerraraFerrara, Italy

**Keywords:** micro-electrocorticography (ECoG), Intracortical microelectrodes, carbon nanotubes, conductive polymers, hydrogel, neural recording, neural stimulation, brain-conformability

## Abstract

Finding the most appropriate technology for building electrodes to be used for long term implants in humans is a challenging issue. What are the most appropriate technologies? How could one achieve robustness, stability, compatibility, efficacy, and versatility, for both recording and stimulation? There are no easy answers to these questions as even the most fundamental and apparently obvious factors to be taken into account, such as the necessary mechanical, electrical and biological properties, and their interplay, are under debate. We present here our approach along three fundamental parallel pathways: we reduced electrode invasiveness and size without impairing signal-to-noise ratio, we increased electrode active surface area by depositing nanostructured materials, and we protected the brain from direct contact with the electrode without compromising performance. Altogether, these results converge toward high-resolution ECoG arrays that are soft and adaptable to cortical folds, and have been proven to provide high spatial and temporal resolution. This method provides a piece of work which, in our view, makes several steps ahead in bringing such novel devices into clinical settings, opening new avenues in diagnostics of brain diseases, and neuroprosthetic applications.

## Introduction

The larger part of our understanding of brain neurophysiology derives from electrical signals experimentally recorded using different types of intracortical penetrating microelectrodes, i.e., multiple-insulated metal microwires (Musallam et al., 2007; Nicolelis and Lebedev, 2009; Marin and Fernández, 2010) or micromachined penetrating microelectrode arrays (Suner et al., 2005; Wise, 2005; Marin and Fernández, 2010) and electrocorticography (ECoG) electrodes (Leuthardt et al., 2004; Kellis et al., 2009; Ritaccio et al., 2011). However, long-term stability is still an issue as regards the clinical application of neural implants, and two parallel avenues of research are currently being explored with a view to resolving this problem. One is the ongoing commitment to improving today's intracortical penetrating microelectrodes, which, despite their superior neural recordings in term of accuracy, bandwidth, and resolution (Leuthardt et al., 2006, 2009), tend to induce acute and chronic inflammatory reactions due to the trauma of mechanical insertion and the tissue response to such a foreign body. Indeed, the latter causes the formation of a glial scar, which distances neurons from the recording sites and thereby impairs signal recording (Schwartz, 2004; Marin and Fernández, 2010). Simultaneously, there is also great effort being expended in the attempt to improve the quality of cortical recording up to a level that could provide sufficient information to decode, for example, motor cortical signals, through the use of very high resolution micro-ECoG arrays (Leuthardt et al., 2004, 2006; Kellis et al., 2009, 2010; Chao et al., 2010).

Although advances have been made, both approaches would greatly benefit from a key improvement, namely reducing the impedance of recording sites. Lowering the impedance is fundamental to enhance the signal recording quality, reduce the background noise, and, consequently, increase the signal-to-noise ratio. At the same time a lower impedance with no dimensional increase enables the injection of relatively large capacitive currents whilst minimizing electrode degradation due to Faradaic effects. Increasing the active surface area of electrodes through the deposition of high-surface-area materials, i.e., nanostructured coatings, has been shown to induce a dramatic drop in impedance (Cui and Martin, 2003; Abidian and Martin, 2008; Green et al., 2008; Keefer et al., 2008; Ferguson et al., 2009; Castagnola et al., 2010; Ansaldo et al., 2011; Baranauskas et al., 2011). This increases their signal-to-noise ratio while enabling electrode miniaturization, which reduces their invasiveness and minimizes tissue damage during insertion. It is expected that very low-impedance and tiny recording sites would also dramatically improve micro-ECoG array technologies, enhancing their sensitivity and spatial selectivity, thereby increasing the information content of the recorded signal. As a consequence, their inter-electrode distance could be reduced, enhancing the spatial resolution (Castagnola et al., 2013a,b).

Another actively pursued key research target is the development of soft, flexible, and conformable electrode substrates, which would reduce implant-related tissue stress. Indeed, the reciprocal movements of brain and skull during behavioral and metabolic processes can generate several negative effects when devices coupled to the bone are *in situ*. However, using a compliant material in penetrating micro-electrodes would help to minimize tissue damage arising from their micro-movements in brain tissue, thereby reducing long-term inflammation and glial scar formation (Rousche et al., 2001; Lee et al., 2004; Takeuchi et al., 2004). In the case of micro-ECoG arrays, the use of a conformable substrate would enable accommodation of both brain curvature and movement, keeping each electrode in the array in intimate contact with the cortical surface and improving the electrical coupling (Hollenberg et al., 2006; Leuthardt et al., 2009; Rubehn et al., 2009; Ritaccio et al., 2011; Khodagholy et al., 2011; Thongpang et al., 2011; Castagnola et al., 2013b). We set out to tackle all of these issues through the application of soft and high-surface-area material coatings to both intracortical and epicortical microelectrodes. We describe the use of a variety of such nanomaterial coatings, documenting their effects on impedance, charge transfer capability, signal-to-noise ratio, and signal recording quality of different types of miniaturized intracortical microelectrodes and very high-resolution micro-ECoG arrays. We also present a validated pragmatic approach to solving the biocompatibility issues and concerns raised by the use of nanomaterial-containing coatings, namely encapsulating nanocoated neural devices with a human fibrin hydrogel layer, which creates a mechanically stable barrier between the nanomaterials and the brain. Finally, we present our method of reducing the invasiveness and increasing the versatility of intracortical microelectrodes and micro-ECoG arrays, making them smaller, softer, and more flexible by exploiting a combination of nanocoatings and polymer-based circuits^1^.

The results we present, in our opinion, provide a method which makes several steps ahead in bringing such novel devices into clinical settings, opening new avenues in diagnostics of brain diseases, and neuroprosthetic applications.

## High-surface-area nanocoatings

The impedance of electrodes is one of the main parameters influencing their suitability for both brain recording and stimulation. Assuming their metal surface is smooth and stable, the charge that can be exchanged with a saline solution, i.e., the current that can flow through it, is limited by the maximum voltage that can be applied without causing irreversible chemical reactions such as electrolysis. In the absence of Faradaic reactions, the metal interface will become charged, meaning that, practically speaking, the electrode must behave like a capacitor to prevent undesirable chemical reactions in the intracellular medium. One way to meet this requirement, which is particularly relevant for purely stimulating electrodes, is to introduce an active material that produces a controlled, reversible and rapid Faradaic reaction (pseudo-capacitive), for instance activated iridium oxide (Cogan et al., 2004; Gawad et al., 2009). A complementary approach, suitable for both stimulating and recording electrodes, would be to increase the electrode capacitance whilst containing its geometric size. This can be achieved, even when using electrochemically inert interfaces, by increasing the electrode surface area by augmenting its roughness/porosity. This, in turn, can be achieved by coating even commonly used metal electrodes with nanostructured high-surface-area (HSA) materials. Indeed, several HSA coatings made from a variety of nanostructured materials have been widely investigated as a means of enhancing the surface properties of traditional metal electrodes, decreasing their impedance, and increasing their charge transfer capability. These include platinum black (Robinson, 1968; Desai et al., 2010), various conductive polymers such as polypyrrole (PPy), polyaniline (PANI) and poly-[3,4 ethylenedioxythiophene] (PEDOT), (Cui et al., 2001; Cui and Martin, 2003; Yang et al., 2005; Ludwig et al., 2006; Kotov et al., 2009), and their carbon nanotube (CNT)-based nanocomposites (Keefer et al., 2008; Castagnola et al., 2009, 2013a,b; Ferguson et al., 2009; Jan et al., 2009; Lu et al., 2010; Ansaldo et al., 2011). As it would be preferable to record and stimulate using the same device, we set up a variety of CNT deposition methods, obtaining coatings to be tested in some of the most demanding electrode applications, i.e., intracortical and flexible epicortical neural recording and stimulation. By electrochemical deposition we were able to coat electrodes made from different metals with PEDOT-CNT, PPy-CNT, and Au-CNT nanostructured coatings. Using an *ad-hoc* process based on catalytic chemical vapor deposition (CVD), we synthetized CNTs directly on metal electrodes able to withstand high temperature (>600°C). It should be noted at this point that, although in the literature all CNT-containing composites (Keefer et al., 2008) are generally referred to as “CNT electrodes,” only in few cases CNTs act as the ultimate electrochemical interface. In most instances, CNTs are mainly used to induce the surface nanostructuring, and, as they are encased in composites, the electrode-solution interface involves no graphitic carbon, but instead conductive polymers (CPs) or metals. In fact, similar results to these so-called “CNT electrodes” could be obtained by adding other additives, such as polyethylene glycol (PEG) or agar, to the deposition solutions, in place of CNTs, to promote HSA formation. However, CNTs are preferred because their high mechanical strength, good electrical properties, high specific area, and high aspect ratio confer composites with superior electrical conductivity and mechanical properties (Green et al., 2008; Gerwig et al., 2012). The main advantage in using electrochemical deposition for coating the electrode surface is that it can be carried out at room temperature, as opposed to *in situ* CNT synthesis, which requires high temperature. This means that it can be applied to a virtually unlimited variety of materials and devices. Conversely, when a direct interaction between CNTs and neural cells is allowed, it has been shown *in vitro* that the intimate contact achieved at this interface gives rise to an excellent electrical coupling (Mazzatenta et al., 2007; Shoval et al., 2009; Sorkin et al., 2009), hinting at a special affinity of exposed CNTs for neural tissue.

### Electroplated HSA coatings

#### Deposition procedure

In the case of CP-CNT composites, polymer and CNT nanocomposites were co-electrodeposited from an aqueous suspension of 1 mg ml^−1^ multi-wall carboxylated CNTs (COOH-MWCNTs, NC 3151, <4% of -COOH functional groups, Nanocyl) containing 0.5 M of the corresponding monomer, 3,4-ethylenedioxythiophene (EDOT, Sigma-Aldrich) or pyrrole (Py, Sigma-Aldrich), and 0.4 wt% of poly(sodium 4-styrene sulfonate) (PSS, Sigma-Aldrich). COOH-MWCNTs were suspended in ultrapure water (Milli-Q, Millipore, USA) via horn sonication (6 s, 66% duty cycle pulses, 4 W ml^−1^, for 30 min) while cooling in an ice bath. PSS and monomers were added to the suspension immediately afterwards, and the solution was kept deoxygenated by bubbling with nitrogen. The electrochemical deposition was carried out in an inert atmosphere in the potentiostatic mode. The polymerization potential was set to 0.55 V vs. Ag/AgCl reference electrode for PPy, and 0.8 V vs. Ag/AgCl reference electrode for PEDOT. For CP-agar coatings, the COOH-MWCNT suspension was replaced by 0.1 wt% agarose. PSS and monomer were added to the stirred solution before it jellified while cooling in an ice bath. Au-CNT nanocomposites were co-electrodeposited by applying monophasic voltage pulses (0.2–1.0 V, 240 s, duty cycle 50%), starting from a 10 mM potassium dicyanoaurate(I) (Sigma-Aldrich) aqueous solution containing 1.5 mg ml^−1^ of partially dispersed MWCNTs (NC 3100, Nanocyl) or 1.5 mg·ml^−1^ of partially dispersed SWCNTs (Cheaptubes). For Au-agar coating the CNTs were replaced by agarose (0.1 wt%).

#### Electroplated HSA coating benchmarking

We compared the electrochemical performance of different HSA coatings using identical planar 3.1 mm^2^ gold and platinum electrodes as benchmarks. The electrochemical behavior of the microelectrodes was studied in a 0.9% sodium chloride (NaCl) aqueous solution, by both cyclic voltammetry (CV)—to quantify their capacitive charging—and electrochemical impedance spectroscopy (EIS)—to determine the electrical properties of the system over a large frequency range. During the CV tests, the working electrode potential was swept between 0.5 and −0.5 V or 0.6 and −1 V vs. Ag/AgCl, maintaining a scan rate of 100 mV/s. During the EIS measurements, a sine wave (10 mV RMS amplitude) was superimposed onto the open circuit potential while varying the frequency from 10^5^ to 1 Hz.

All electrochemical depositions were carried out using a potentiostat/galvanostat (Parstat 2273, Princeton Applied Research), while a potentiostat/galvanostat/ZRA (Reference 600, Gamry Instruments, USA) was used for electrochemical characterization. The electrochemical cell was a three-electrode cell. A platinum wire was used as the counter electrode and an Ag/AgCl electrode was used as the reference electrode.

**Figure 1:**
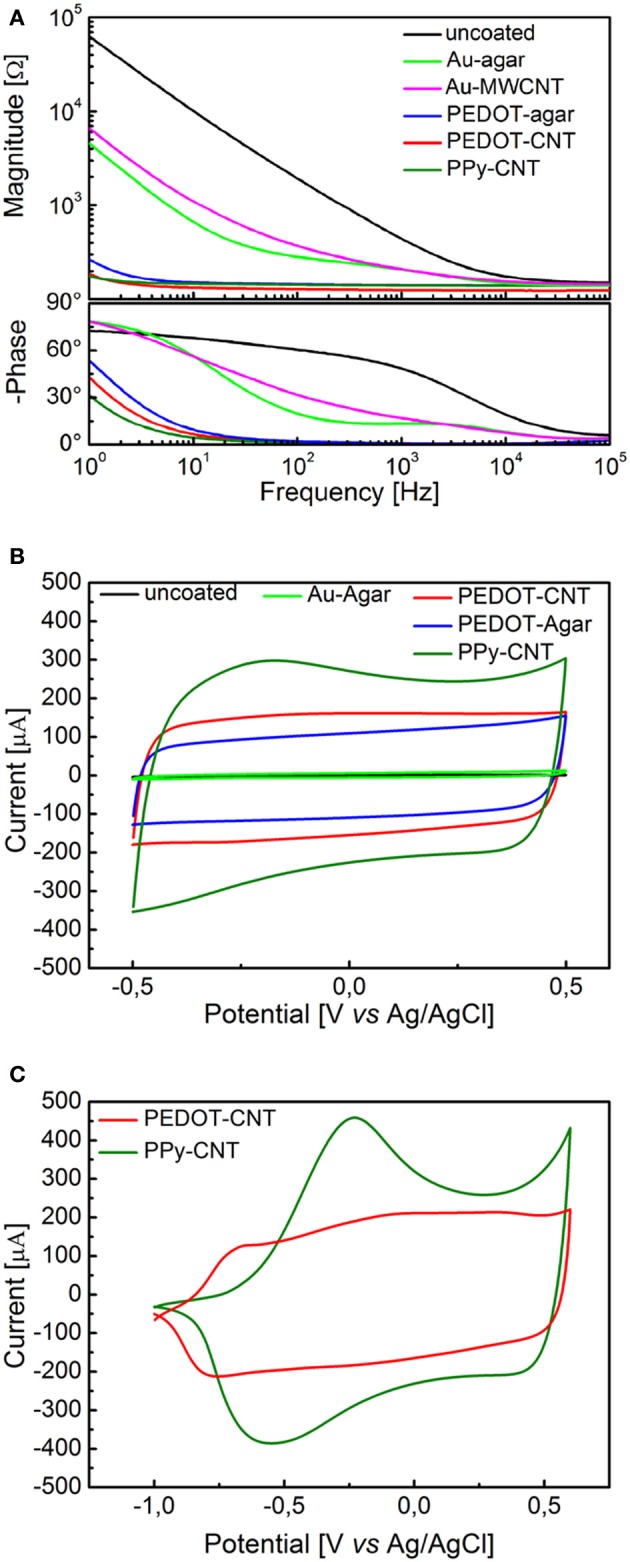
**(A)** Impedance spectra of a 0.031 cm^2^ Pt electrode, uncoated (black) and with electrodeposited Au-MWCNT (pink), Au-agar (green), PPy-CNT (dark green), PEDOT-agar (blue), or PEDOT-CNT (red). **(B)** Cyclic voltammograms between −0.5 and 0.5 V of a 0.031 cm^2^ Pt electrode, uncoated (black), or with electrodeposited Au-agar (green), PPy-CNT (dark green), PEDOT-agar (blue), or PEDOT-CNT (red). **(C)**, Cyclic voltammograms between −1.0 and 0.6 V of a 0.031 cm^2^ Pt electrode coated with PPy-CNT (dark green) and PEDOT-CNT (red).

Figure 1A presents a comparison between the EIS spectra of identical planar platinum electrodes uncoated or coated with PPy-CNTs, PEDOT-CNTs, PEDOT-agar, Au-CNT, and Au-agar, respectively. In all cases the potentiostatic deposition of the CP composites was performed applying a charge density of 700 mC/cm^2^. All nanocoatings tested resulted in a reduction in impedance across the entire frequency range (1–10^5^ Hz). The impedance magnitude values at 100 Hz and 1 kHz are reported in Table 1, together with the corresponding total charge transfer capability (CTC_tot_) values. The CTCs were calculated as the time-integral of an entire CV cycle between 0.5 and −0.5 V (CTC_tot_1), or between 0.6 and −1V (CTC_tot_2). Typical CVs measured for differently coated electrodes are reported in Figure 1B (0.5 to −0.5 V range) and Figure 1C (0.6 to −1.0 V range for PPy-CNT- and PEDOT-CNT-coated electrodes). In both cases it can be observed that when the same charge density is applied during electrodeposition, the CTC_tot_ of the PPy-CNT coating is greater than that of the PEDOT-CNT coating, but that the peak potentials of the PEDOT composites are about 200 mV more negative than those of the PPy composite (Figure 1C), extending the PEDOT capacitive behavior over a wider potential window. Moreover, the PEDOT-CNT coating provided the lowest impedance at all frequencies above 2 Hz.

**Table 1:** **Electrochemical characterization of HSA coatings**.

	**|Z| @ 100 Hz [Ω]**	**|Z| @ 1 kHz [Ω]**	**CTC_tot_1[mC cm-2]**	**CTC_tot_2[mC cm-2]**
PEDOT-agar	142.6 ± 1.7	139.5 ± 1.6	149.7 ± 7.3	246.2 ± 4.9
PEDOT-CNT	127.6 ± 0.1	125.0 ± 0.2	92.3 ± 4.6	158.7 ± 8.2
PPy-CNT	138.7 ± 4.3	136.7 ± 4.1	67.7 ± 0.5	118.9 ± 4.8
Au-agar	291 ± 12	214.6 ± 8.3	3.1 ± 0.7	6.2 ± 0.5
Au-CNT	334 ± 50	192 ± 25	2.5 ± 0.3	4.6 ± 0.2
Uncoated	1915 ± 203	441 ± 7	0.5 ± 0.1	3.3 ± 0.2

### Procedure for chemical vapor deposition of carbon nanotubes

In order to selectively synthetize CNTs on the metal tip of an electrode of arbitrary shape, we devised a nickel catalyst deposition method based on electrode electroplating, rather than relying on the more common physical deposition methods (such as dip-coating, spray-coating, evaporation, or sputtering). Nickel was electroplated from an aqueous solution containing 300 g l^−1^ nickel(II) sulfate hexaydrate (NiSO_4_·6H_2_O, CAS 10101-97-0, *puriss*. p.a. Riedel-de Haën), 90 g l^−1^ nickel chloride hexaydrate (NiCl_2_ 3 6H_2_O, CAS 7791-20-0 *purum*, p.a.; >99.0% Fluka), and 45 mg l^−1^ boric acid (H_3_BO_3_, CAS 10043-35-3, *puriss*. p.a. Riedel-de Haën). Electrodeposition was performed in the potentiostatic mode at −0.9 V vs. an Ag/AgCl reference electrode. The depositions were carried out in an inert atmosphere at 45°C and pH 4. The amount of catalyst deposited onto the electrode was determined by controlling the charge passed during the deposition. The CVD process was performed in a quartz tube reactor, 120 cm long and 25 mm in diameter, passing through a Lenton PSC 12/--/600H split furnace. The catalyst was activated by flowing a forming gas (Ar 95 sccm, H_2_, 5 sccm, ambient pressure) while heating the samples up to 650°C; the carbon feedstock was then introduced into the reaction chamber by bubbling the same gas mixture through a Drechsel washing bottle with glass filter disk, filled with absolute ethanol at room temperature. Reaction time was 15 min at 650°C.

## Application of nanocoatings

### Intracortical platform

#### Reduced impedance and increased charge transfer capability

**Figure 2:**
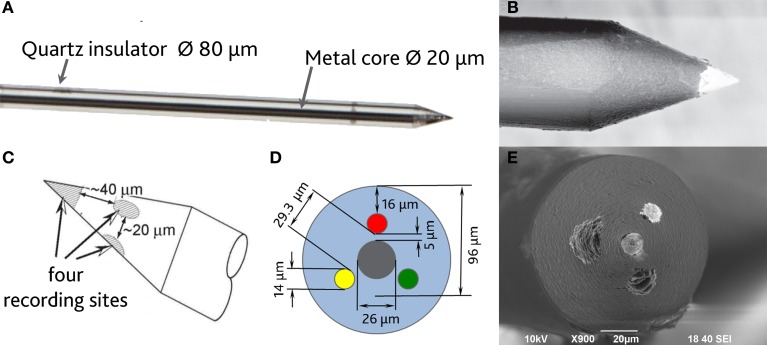
**(A)** Optical image of a single sharp microelectrode. **(B)** Scanning electron micrograph of a single sharp microelectrode. **(C,D)** Schematic datasheet of a 4-core quartz-platinum/tungsten tetrode microelectrode (Thomas Recording, Giessen, Germany). **(E)** Tetrode scanning electron micrograph (s.e.m.).

**Figure 3:**
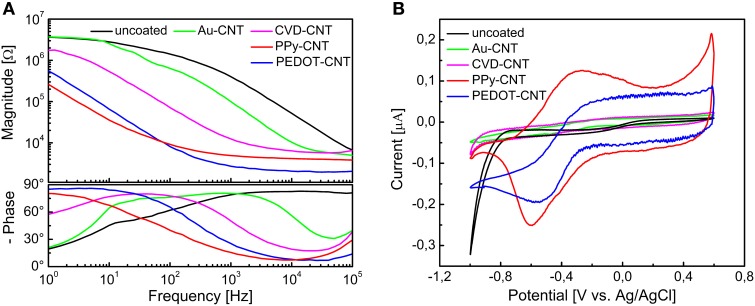
**(A)** Impedance spectra of a single intracortical electrode, uncoated (black), or coated with Au-CNT (green) PPy-CNT (red), or PEDOT-CNT (blue), electrodeposited and CNT coated by direct growth with CVD. **(B)** Cyclic voltammograms of a single intracortical electrode, uncoated (black) or coated with Au-CNT (green), PPy-CNT (red) or PEDOT-CNT (blue), electrodeposited, and CNT coated by direct growth with CVD.

**Figure 4:**
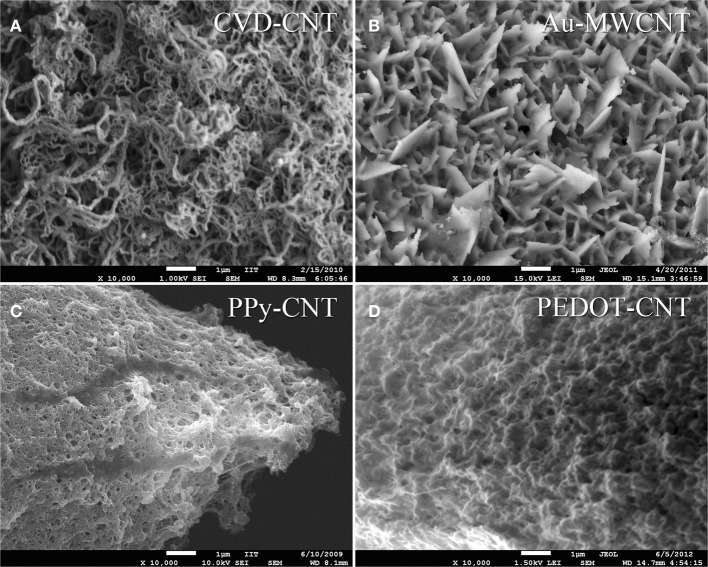
**Scanning electron micrograph of (A) as grown CNT-CVD coatings, (B) Au-MWCNT coating, (C) PPy-CNT coating, (D) PEDOT-CNT coating**.

**Figure 5:**
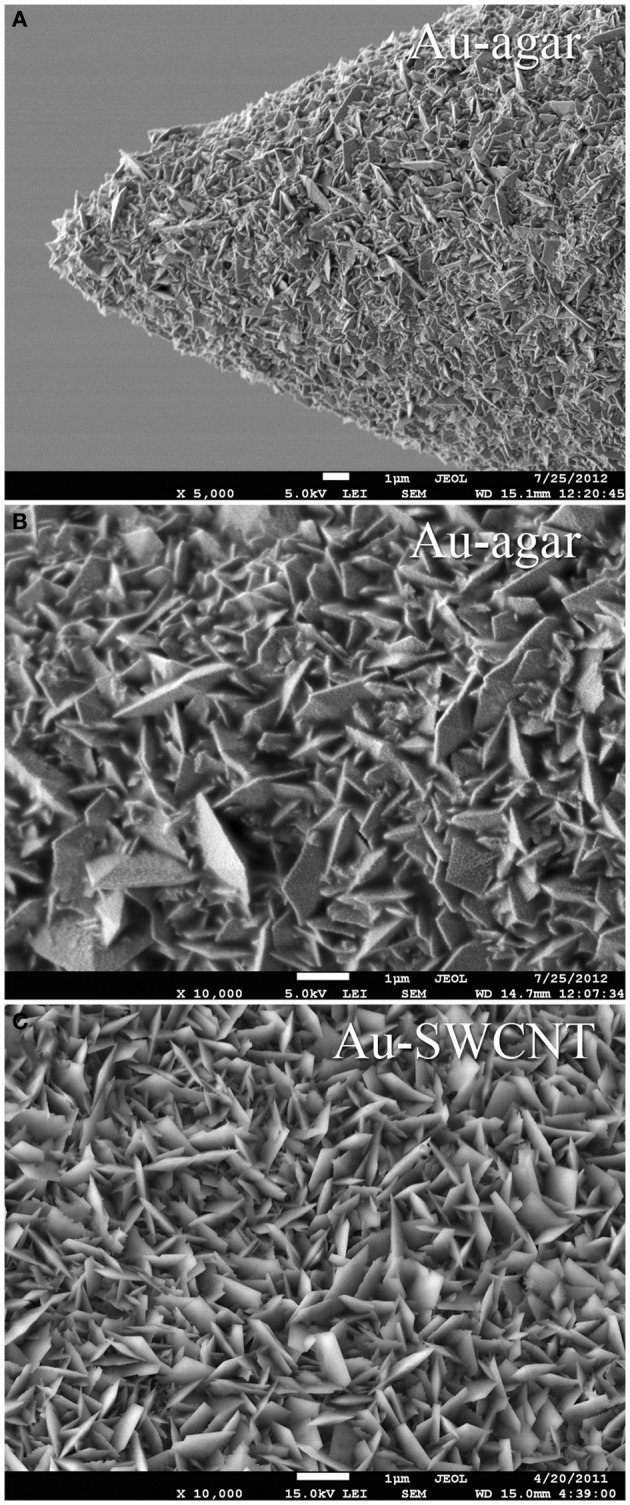
**Scanning electron micrograph of (A) a single tip Au-agar coated microelectrode, (B) Au-agar coating, (C) Au-SWCNT coating**.

In order to be able to compare all the previously described HSA coatings, including the CNTs directly synthetized on the electrode tip at high temperature, we selected as an intracortical electrode platform quartz glass insulated platinum/tungsten wires of 80 μm outer shank diameter and 20 μm metal core diameter (Thomas Recording, Giessen, Germany). We prepared and characterized identical electrodes coated with *in situ* synthetized CNTs (CVD-CNT) and with different electrochemically co-deposited composites, namely Au-CNT, Au-agar, PPy-CNT, PEDOT-CNT, and PEDOT-agar. The insulated wire was ground using a grinding machine (DIECKL-ST, Thomas Recording), thereby providing sharp tips with a typical geometric area of about 1000 μm^2^ and an impedance magnitude ranging from 0.5 to 0.7 MΩ at 1 kHz. We used both of these single electrodes (Figures 2A,B reports optical and SEM images respectively) and 4-core quartz-platinum/tungsten microelectrodes (tetrodes) having similar electrical properties to perform direct comparisons of the coatings' performance in simultaneous neural recordings. The corresponding datasheet schematics from Thomas Recording are shown in Figures 2C,D. A SEM picture of the tetrode is shown in Figure 2E. Electrochemical characterizations of the typical uncoated (black), electroplated Au-CNT (green), PPy-CNT (red) and PEDOT-CNT (blue), and CNT-CVD coated (magenta) electrodes were performed using impedance spectroscopy (Figure 3A), and cyclic voltammetry between 0.6 and −1.0 V vs. Ag/AgCl (Figure 3B). The electrochemical performance reported here is related to “viable” electrodes, as defined in our previous work (Ansaldo et al., 2011), in which we emphasized that special attention must be paid when presenting “dramatic” changes in impedance and charge transfer capability in electrodes designed for intracortical neural recording and stimulation. Indeed, although electrode impedance could feasibly be lowered by many orders of magnitude by increasing the amount of material, thick coatings are usually mechanically unstable. Here we consider “viable” electrodes those capable of withstanding dura mater penetration, i.e., displaying no significant changes in appearance or electrochemical performance even after an acute intracortical recording session (Ansaldo et al., 2011). According to this definition, our viable CNT-CVD-coated electrodes typically had an impedance of 16.3 ± 4.8 kΩ at 1 kHz, with a 70.8 ± 1.1 mC·cm^2^ CTC_tot_ (Castagnola et al., 2010; Ansaldo et al., 2011), a significant improvement with respect to the uncoated electrodes (typically 600 kΩ and 20 mC cm^−2^ CTC_tot_). In the case of PPy-CNT and PEDOT-CNT coatings, viable electrodes were consistently produced by using charge densities of 0.6 C cm^−2^ during deposition. Impedance magnitude and CTC_tot_ were 5.7 ± 0.3 kΩ @1 kHz and 212.3 ± 23.9 mC cm^−2^, respectively, for PPy-CNT-coated microelectrodes, and 4.5 ± 0.6 kΩ @1 kHz and 180.5 ± 40.2 mC cm^−2^ for PEDOT-CNT-coated microelectrodes. In the case of Au-CNT coatings, improvements were limited by the intrinsic behavior of gold, which produces nanorough lamellar surfaces rather that nanoporous ones. The best values obtained for viable electrodes were an impedance of 59.6 ± 11.2 kΩ @1 kHz and a CTC_tot_ of 65.6 ± 13.6 mC cm^−2^ (Ansaldo et al., 2011). CTC_tot_ and CTC_tot_2 were calculated as previously described. The reduction in impedance achieved depended on the coating, its nature and morphology, but all appeared to pave the way to a better signal-to-noise ratio in both single-neuron and multiunit recordings. The corresponding increase in CTC also makes these electrodes suitable for both recording and stimulation. The morphology of the different coatings can be appreciated by looking at the SEM pictures of grown CNT-CVD (Figure 4A), Au-CNT (Figure 4B), PPy-CNT (Figure 4C), and PEDOT-CNT (Figure 4D) electrodes. Nanorough lamellar coatings, similar to Au-CNT, can be also obtained by exploiting gold co-deposition in the presence of agarose gel (Figures 5A,B), or SWCNTs (Figure 5C).

**Figure 6:**
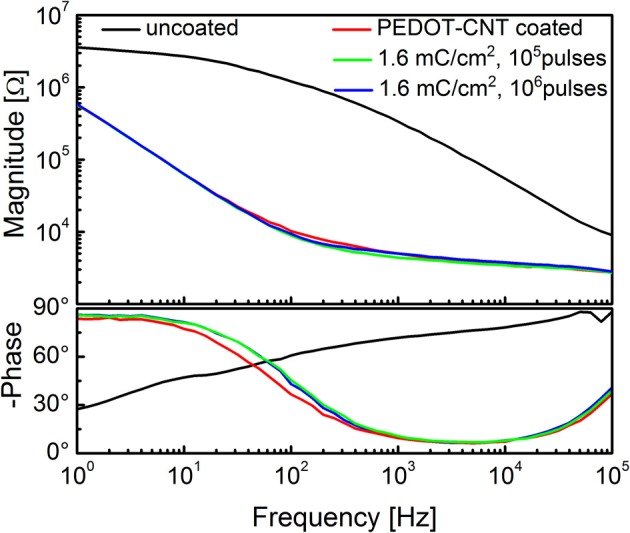
**Impedance spectra of a single intracortical electrode uncoated (black), and PEDOT-CNT-coated (red) and after stimulation experiments**.

To verify whether these coated microelectrodes are capable of stimulating the brain cortex, we estimated the charge injection limit for a set of typical electrodes, using cathodic-first charge-balanced biphasic symmetrical current pulses (Brummer et al., 1983; Cogan, 2008). The charge injection limit (Q_inj_), defined as the maximum quantity of charge an electrode can inject before reaching the water electrolysis potential, was calculated as the time-integral of the current in the loading phase normalized by the geometric area of the uncoated microelectrode; water electrolysis potentials were determined experimentally via cyclic voltammetry. We used pulses having a total period of 2 ms and a cathodic duration of 500 μ s (Ansaldo et al., 2011). To establish whether these electrodes could be used for long-term (chronic) neural stimulation and recording, we tested the stability of the different coatings under prolonged stimulation, and then compared the impedance of typical viable electrodes before and after applying a series of cathodic-first charge-balanced biphasic current pulses. The pulse duration was varied between 2 and 3 ms, the charge density between 0.4 and 1.6 mC cm^−2^, and the cathodic pulse duration between 100 and 500 μs. The charge density applied was chosen according to the Q_inj_ for each material, namely 7 mC cm^−2^ for PPy-CNT microelectrodes, 4 mC cm^−2^ for CVD-CNT microelectrodes, and 0.8 mC cm^−2^ for Au-CNT microelectrodes. Even the relatively small Q_inj_ of Au-CNT electrodes is more than double that of the uncoated electrodes (0.2–0.4 mC cm^−2^). As regards stability over time, CVD-CNT-coated electrodes were found to be stable, and could withstand a million 1.6 mC cm^−2^ pulses without any degradation. PPy-CNT coatings rapidly degraded after a few thousand pulses at 1.6 mC cm^−2^, while Au-CNT composite coatings were nearly as stable as CVD-CNTs, also being able to resist a million pulses, although their charge density had to be limited to 0.8 mC cm^−2^ in order to prevent Faradaic reactions (see Ansaldo et al., 2011). We can therefore conclude that CNTs selectively synthetized directly on the tip of neural microelectrodes by CVD are able to outperform the other so-called “CNT coatings” laid down by electrodeposition of CNT composites. Indeed, the CNT-CVD coating not only reduced microelectrode impedance, but was also able to withstand the repeated high-current pulses required for neural stimulation without degrading. This and its stability over time make it an excellent candidate for improving electrodes for use in chronic applications, especially when both neural recording and stimulation are required. Indeed, the only foreseeable limitation of CNT-CVD coatings resides in the high temperature (650°C) required for their deposition. Analysis of the PEDOT-CNT coating also yielded interesting results. In fact, it has already been reported in various studies that PEDOT may be a viable alternative to PPy for chronic, long-term implantations (Cui and Martin, 2003; Yang et al., 2005; Ludwig et al., 2006), as it is electrochemically more stable than PPy (Yamato et al., 1995). We measured a charge injection limit of 7 mC cm^−2^ on our PEDOT-CNT-coated microelectrodes. While this is identical to the charge on PPy-CNT-coated electrodes, PEDOT-CNT versions are able to withstand a million 1.6 mC cm^−2^ pulses without significant degradation, as demonstrated by the impedance spectra of a typical PEDOT-CNT coated electrode, reported in Figure 6. This suggests that PEDOT-CNT coatings are also excellent candidates for chronic applications, especially when both neural recording and stimulation have to be performed and a room-temperature deposition technique is required. Indeed, an initial validation of the suitability of these microelectrodes for *in vivo* use has already been obtained through acute intracortical recording sessions in rats (Ansaldo et al., 2011; Baranauskas et al., 2011).

#### CNT-composite coating improves multiunit signal-to-noise ratio of neural microelectrodes

Although it seems safe to assume that a low-impedance electrode has lower noise and is able to record larger signals, in fact the relationship between its resistivity and capacitance, and its ability to provide high-quality recordings are not straightforward. Indeed, the *in vivo* performance of an electrode is strongly influenced by many features of its surrounding environment (the brain), as well as the geometric area of the electrode (with respect to the size of the cells, i.e., the signal sources), and the presence of background biological activity, which remains too low to be properly detected, but contributes to the neural signal (the so-called biological noise) and therefore cannot be ignored. While the impedance reduction has a well-known impact on the Johnson-Nyquist noise (the thermal noise), any background signal that cannot be clearly isolated as neural activity will significantly contribute to total noise of a low-impedance electrode. Moreover, in the case of HSA-coated electrodes, the high specific capacitance, combined with the relatively high transversal resistivity of the coating, will significantly lower the speed of the signal on the electrode surface, meaning that the geometric size of the electrode can influence the frequency response at relatively low frequencies.

In order to investigate the effect of the very low impedance of CNT-based HSA coatings on signal acquisition, we devised a series of specific experiments using PPy-CNT coated electrodes to perform “acute” recording sessions on rats (Baranauskas et al., 2011). We studied in detail the impedance, the thermal, electronic and *in-vivo* noise, and the neural signals using ten single microelectrodes, PPy-CNT coated and uncoated, and two tetrodes, two of whose recording sites were coated with CNT-PPy while the other two remained untreated. This experiment enabled us to directly compare very closely positioned coated and uncoated electrodes during the same recording session.

As pointed out previously, during an *in vivo* recording session, neural signals are affected by several noise sources of both biological and non-biological origin. The non-biological sources include the electronic noise created by the amplifier, the thermal noise (Johnson-Nyquist), and the double-layer noise at the interface between the solution and microelectrode surface. The biological noise also includes additional thermal noise, created by the presence of brain tissue, and signals emitted by distant neurons. These neural signals are too small to be usefully distinguished, and therefore behave as noise for the majority of neural signal analyses (Harris et al., 2000; Quiroga et al., 2004; Ferguson et al., 2009). In the case of our electrodes, the non-biological noise contribution—including recording system noise, and filtering—was estimated by recording the noise signals from single microelectrodes immersed in a saline solution (NaCl 0.9%). In the case of PPy-CNT coated electrodes, we detected reduced noise over the entire useful frequency range (250–8000 Hz), and, in particular, in the range of frequencies corresponding to the multi-unit neural activity (MUA, ~150–1500 Hz). In the 250–8000 Hz frequency band, the overall noise measured was reduced by ~30%, from 5.9 ± 0.2 μ V rms to 4.2 ± 0.3 μ V rms. This difference was significant (*p* < 0.015; one-tailed *t*-test).

As discussed previously, the background noise is higher in the brain due to biological sources. Hence, in order to evaluate the biological noise contribution, we recorded from the deep layers of the primary somatosensory cortex (S1) in rats anaesthetized with Zoletil (30 mg/kg) and Xylazine (5 mg/kg). Indeed, under these conditions S1 switches between periods of high neural activity (“bursts”) and periods of much lower activity (“pauses”). These periods of high and low activity are synchronized across large brain areas (>1 mm), and during the “pauses” the noise contribution of distant neuronal activity is minimal (Erchova et al., 2002), but during “bursts” it is possible to evaluate the background noise caused by the maximal activity of such neurons. In order to eliminate the effect of the presence of these burst spikes, the noise amplitude was estimated using a median value (Quiroga et al., 2004). We demonstrated that during “pauses,” when the neuronal activity was low, the observed noise reduction ranged between 30 and 40%, comparable to data reported in the literature (Gabay et al., 2007; Keefer et al., 2008) though smaller than that of the saline test (55%). In contrast, during “bursts” the increased sensitivity provided by the PPy-CNT coating resulted in a larger contribution from distant neuronal activity, and a slight uptrend was observed from 11.2 ± 3.0 μ V rms (uncoated) to 13.8 ± 4.0 μ V rms (CNT-PPy, *p* < 0.2).

Moving on to the analysis of recorded neural signals, we estimated the amplitude for single spikes, local field potentials (LFP) and MUAs (Baranauskas et al., 2011). Tests were carried out using platinum/tungsten tetrodes featuring two PPy-CNT-coated and two uncoated recording sites. Although the average spike amplitudes of the two types of electrodes were similar (0.45 ± 0.03 mV vs. 0.40 ± 0.03 mV), the maximal spike amplitude was greater with the coated versions (increased from 0.52 ± 0.03 mV to 0.75 ± 0.04 mV). Moreover, more neuronal units were detected using the PPy-CNT coated electrodes (22 with respect to 16 units), as these were able to detect signals arising from more distant neurons in which smaller spikes dominate. This ability to detect a certain number of weaker signals may explain why the increase in maximal recorded amplitude was not accompanied by a similar improvement in average, further suggesting that average spike amplitude cannot be assumed as a reliable indicator of recording ability.

The contribution of MUA to the overall spectral power density (SPD) was estimated by subtracting the SPD of the signal recorded during “bursts” from the one recorded during “pauses.” A significant increase in the SPD of the signals recorded using the PPy-CNT-coated electrode in the frequency range 200–1000 Hz was observed. Notably, analysis of the low frequency part of the spectrum (~1–250 Hz) recorded using PPy-CNT-coated microelectrodes revealed a marked reduction—of almost 3 orders of magnitude—in the 50 Hz interference (the frequency of the European electric power network), and total rejection of its higher harmonics. There are at least two reasons why this reduction in 50-Hz noise is particularly important from a neurophysiological standpoint. First, it reduces the risk of amplifier saturation during LFP recordings, and second the power line frequency of 50 Hz precisely matches the middle frequency of the so called “gamma range,” which is thought to reflect a number of important brain functions (Gray et al., 1989; Fries et al., 2007). To sum up, we found that the most important improvements conferred by PPy-CNT coating of microelectrodes can be observed in the frequency range from 150 to 1500 Hz, which mainly corresponds to the multiunit signal and, partially, to the high-frequency end of LFPs. In this frequency range, the non-biological noise power is reduced by up to three-fold, while the neural signal power is increased by up to nine-fold. The combination of lower noise and stronger neural signal resulted in an approximately four-fold improvement in SNR in the middle of this frequency range. Our results also suggest that the coated microelectrodes offer considerable advantages for studying brain signals in the gamma frequency range, thanks to their greater electromagnetic noise rejection.

### Flexible micro-ECoG arrays

**Figure 7:**
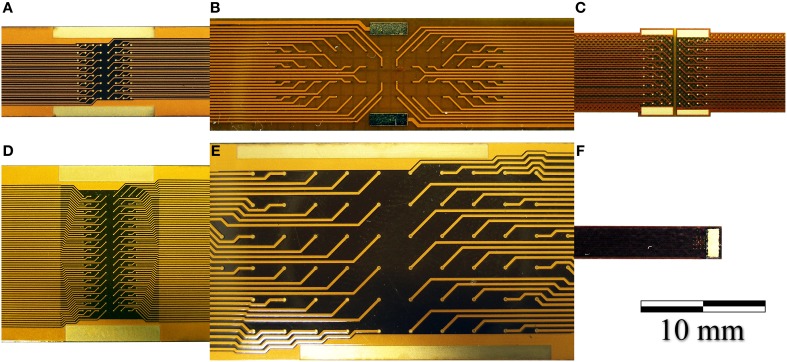
**Photographs of (A) a 64-electrode array with 4.2 × 4.2 mm total recording area and 140 μm diameter recording sites. (B)** A 64-electrode array with 3.6 × 18.0 mm total recording area and 100 μm diameter recording sites; **(C)** A 64-electrode matrix with 5 × 5 mm total recording area, designed for the marmoset; **(D)** A 128-electrode array with 4.2 × 8.4 mm total recording area and 100 μm diameter recording sites; **(E)** A 64-electrode array with 12.5 × 27.5 mm total recording area and 200 μm diameter recording sites. **(F)** A 16-electrode array with 1.2 × 1.2 mm total recording area and 100 μm diameter recording sites.

We developed a family of micro-electrocorticography (ECoG) arrays based on the commercially available flexible printed circuit (FPC) technology, tailoring the design according to the different neurophysiological experiments to be conducted. The choice of a well-established, reliable industrial process greatly reduced the time required to move from concept to prototype, and would likewise simplify production. We developed devices suitable for ECoG on both rats and primates (marmoset), as well as prototypes of devices suitable for the use on humans, having from 16 up to 128 recording sites with electrode diameters ranging from 75 up to 200 μm and an inter-electrode pitch from 300 up to 1.2 mm. Some examples are shown in Figure 7.

**Figure 8:**
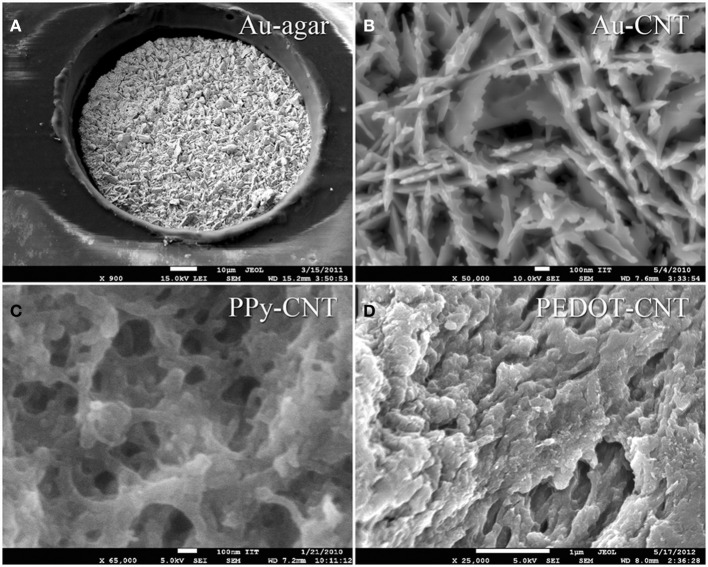
**s.e.m. images of: (A) a 100 μm Au-agar-coated recording site; (B)** Au-CNT coating; **(C)** PPy-CNT coating; **(D)** PEDOT-CNT coating.

The FPCs consist of a double-sided copper circuit (9 μm thick) on a 25 μm polyimide film. After photo etching, the circuit is embedded in two polyimide coverlays (12.5 μm of polyimide plus 12.5 μm of pressure-sensitive acrylic adhesive) by cold lamination. The electrodes are then exposed by laser ablation, and passivated by a 1-μ m thick layer of electroplated gold. The micro-ECoG devices were post-processed in our laboratory by electrochemical deposition of a nanostructured gold layer, which acts as an adhesion layer for PEDOT-CNT or PPy-CNT composites (Castagnola et al., 2013a,b). PEDOT-CNT or PPy-CNT nanocomposites were then co-deposited electrochemically onto the micro-ECoG recording sites to reduce their impedance and increase their charge transfer capabilities. SEM images showing the typical morphologies of these coatings when deposited on the micro-ECOG electrodes are shown in Figure 8.

**Figure 9:**
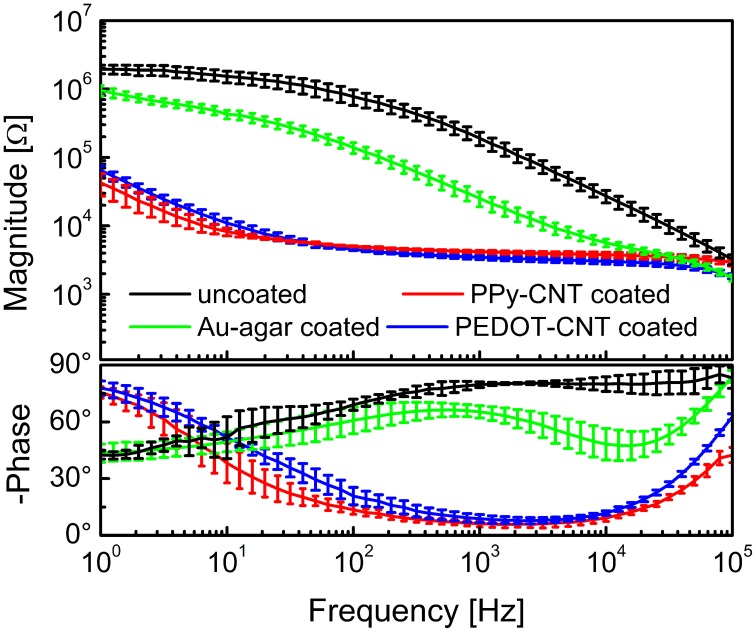
**Impedance spectra of 100-μm-diameter ECoG array recording sites (mean and standard deviation from 64 recording sites each) before (black) and after Au-agar (green), PPy-CNT (red) and PEDOT-CNT (blue) electrodeposition**.

**Figure 10:**
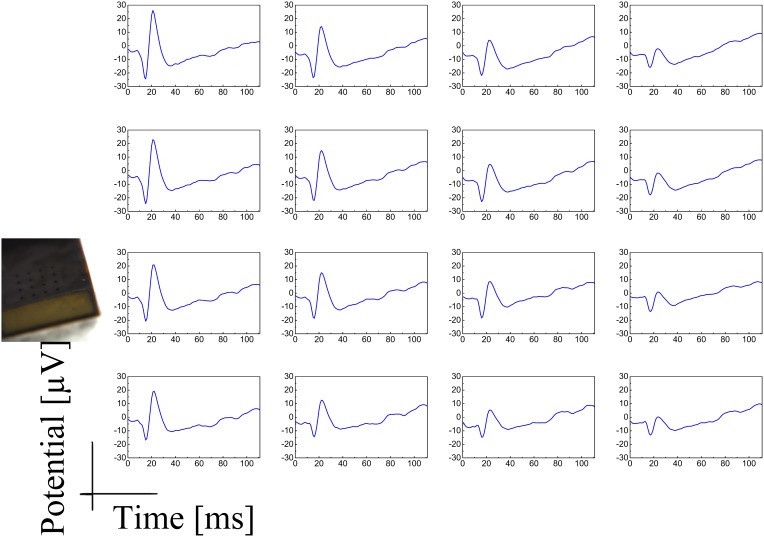
**SEP response of a 4 × 4 micro-ECoG array (inset) with a 1 × 1 mm total recording area, 100 μm diameter recording sites, and 300 μm inter-electrode pitch, coated with PEDOT-CNT to the first 12-ms truncated Gaussian of a 9-Hz train of rat multiwhisker deflections of 0.8° mm**. The data are averaged on 20 repetitions of the stimulation pattern.

Both the Au-CNT and Au-agar coatings present nanorough lamellar structures that reduce the electrode impedance by two-thirds with respect to the uncoated electrode (100 Hz, LFP central band), while the PEDOT-CNT and PPy-CNT composite coatings present a compact, sponge-like morphology, which provides a dramatic reduction in impedance of up to greater than 2 orders of magnitude over the whole frequency range of interest (1 Hz–1 kHz), and especially at 100 Hz. Figure 9 reports the impedance spectra of 100 μm diameter ECoG array recording sites before and after Au-agar, PPy-CNT and PEDOT-CNT electrodeposition. The values shown were obtained by averaging the impedance spectra of 64 electrodes for each material studied. The impedance values (mean ± standard deviation) at 100 Hz were 774.5 ± 198.0 kΩ for the uncoated electrodes, 138.9 ± 24.9 kΩ for Au-CNTs, 5.0 ± 0.1 kΩ for PPy-CNTs, and 4.5 ± 0.2 kΩ for PEDOT-CNTs. It should be noted that our PEDOT-CNT-coated devices are fully compatible with sterilization in saturated water vapor at 122°C and 2 atm for 20 min, one of the most commonly used sterilization processes in hospitals, making them suitable candidates for clinical applications (see Castagnola et al., 2013a,b). In order to establish whether they are suitable for cortical stimulation, we investigated their ability to withstand a series of cathodic-first charge-balanced biphasic current pulses with a 2 ms pulse duration, 500 μ s cathodic pulse duration, and 5.7 mC cm^−2^ charge density, measuring whether there was any change in the impedance spectra after each series. We chose to test PEDOT-CNT-coated micro-ECoG arrays alone, as we know from previous tests that PPy is not electrochemically stable under similar conditions. With our PEDOT-CNT arrays, impedance spectra did not change after four million pulses (data not shown), even when applying a ten times larger charge density, exhibiting the ability to handle the higher currents needed to stimulate neurons from the cortical surface. An example of somatosensory-evoked potentials (SEP) recorded using a 4 × 4 micro-ECoG array (inset) with a 1 × 1 mm total recording area, 100 μm diameter recording sites and 300 μm inter-electrode pitch coated with PEDOT-CNT is shown in Figure 10. The graphs show the response of each single electrode to the first 12-ms truncated Gaussian of a 9-Hz train of a rat multiwhisker deflections of 0.8 mm. The data are averaged from 20 repetitions of the stimulation pattern.

## Reduced invasiveness and increased versatility through nanocoatings and polymer-based circuits: smaller and softer electrodes

### Low-invasive intracortical microspheres

The insertion of any electrode into living brain tissue unavoidably results in a mechanical trauma that activates a cascade of events leading to a neural loss around the implant and its encapsulation in a high-impedance scar made of glial and immune cells, undermining the goal of maintaining long-term communication with neurons (Ward et al., 2009; Marin and Fernández, 2010). However, the physical properties of the whole device,—e.g., its size, shape and stiffness—can play a fundamental role in modulating the tissue reaction, and can be altered to reduce the insertion trauma and to aid accommodation of the large stiffness mismatch between the device and the nervous system tissue (Edell et al., 1992; Ward et al., 2009; Marin and Fernández, 2010). Aside from coating the electrodes with different biomolecules, anti-inflammatory compounds, hydrogels (Crompton et al., 2007; Zhong and Bellamkonda, 2007; Abidian and Martin, 2009), polymers (Ludwig et al., 2006; Leung et al., 2008; Grill et al., 2009) or carbon nanotubes (Lovat et al., 2005; Mazzatenta et al., 2007), another strategy is to modify the physical dimensions, and geometry of the neural probe (Stice et al., 2007; Ward et al., 2009).

Our approach was to minimize the physical size of the probe while maximizing the flexibility of the tether between the electrode and the connection to the external recording/stimulation system in order to be able to accommodate movements and motions of the brain with respect to the skull. As the smaller the size of the electrode, the greater the impedance, we endeavored to exploit the majority of the available surface of the probe, abandoning the standard shapes, which usually expose either flat or conical electrode surfaces. Instead we developed a process in which we created a conductive sphere (the electrode) that is much larger in diameter than the wire that connects it to the external recording chain. In fact, while the surface area of the electrode is crucial for recording and stimulation, even a very thin metallic wire used as connector will have a much lower impedance than that of the electrochemical interface. Another advantage of the spherical shape is that the electrode does not need a rigid stem, as it can be kept in position by the hydrostatic pressure of the brain.

To demonstrate this concept, we grew microspheres of fuzzy gold at the end of 12-μm polyimide-insulated Pt wires by electrochemical deposition, starting from a 10 mM potassium dicyanoaurate(I) agar gel (0.1 wt%), and applying monophasic voltage pulses (0.2–1.0 V, 240 s, duty cycle 50%). The temperature of the gel was kept at 45°C.

**Figure 11:**
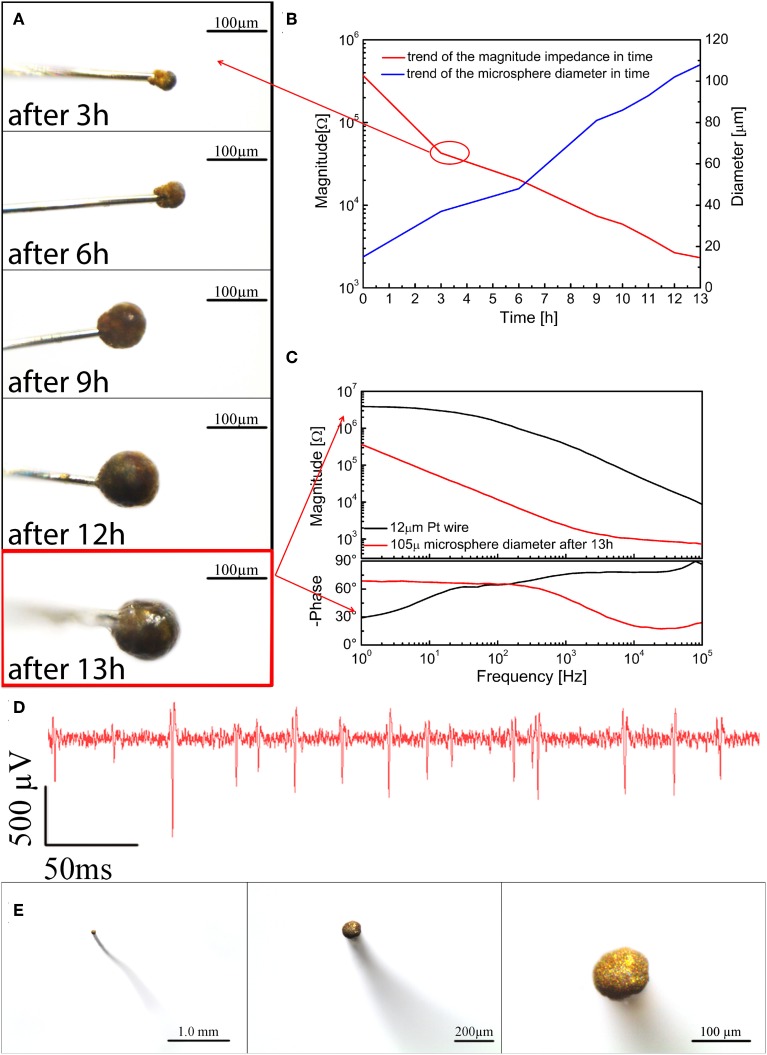
**(A)** Optical images of the microspheres after 3, 6, 9, 12, and 13 h of electrodeposition; **(B)** impedance magnitude at 1 kHz and diameters of the microspheres after 3, 6, 9, 12, and 13 h of electrodeposition; **(C)** Impedance after 13 h of electrodeposition; **(D)** an example of spike activity recorded using a 100 μm microsphere; **(E)** from left to right different top view optical magnifications of a typical microsphere.

We characterized our microspheres both optically (Figure 11A) and electrochemically, measuring the size and impedance (Figure 11B) after 3, 6, 9, 12, and 13 h of electrodeposition. After the first 3 h, a huge reduction in impedance was observed, which can be linked to both the increased electrode size and the far greater surface area generated by the microstucturing. Over the following 10 h the impedance decreased by another order of magnitude, with a fairly linear trend in good accord with its increase in diameter. After 13 h we reached a diameter of 105 μm, and the corresponding impedance was less than 1 kΩ at 1 kHz. The impedance spectrum of a 105-μm diameter microsphere is shown in Figure 11C. An example of a signal trace recorded using a 100 μm diameter microsphere is shown in Figure 11D. The very thin Pt wire is extremely flexible and, once the microsphere is inserted, bends easily, reducing the mechanical stress on the surrounding tissue, while the sphere, as mentioned before, remains in place thanks to the hydrostatic pressure of the brain. The change in diameter of the microsphere can be better appreciated in Figure 11E.

### Ultra-flexible and brain-conformable micro-ECoG device with low-impedance microelectrodes

**Figure 12:**
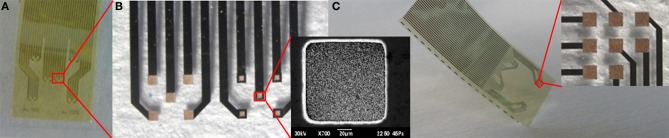
**(A)** One possible ultraflexible micro-ECoG array layout. **(B)** Details of the two groups of five recording sites 200° × 200° μm and 100° × 100° μm in size, and a scanning electron micrograph of a 100 × 100 μm Au recording site (inset). **(C)** Other possible ultraflexible micro-ECoG array layouts and detail of a group of eight 200 × 200° μm recording sites (inset).

Although our flexible ECoG arrays were able to record brain activity in acute sessions, a long-term large-area implant comprising a high-resolution ECoG array would ideally require more flexible and brain-conformable devices that can accommodate brain curvature, compliantly covering neighboring gyri and crossing sulci while keeping the electrodes in intimate contact with the brain cortex. One way of attempting to meet this requirement is to reduce device thickness by developing appropriate technologies. To this end we modified prototypes of gold-on-polyimide ultra-flexible micro-ECoG arrays. The fabrication procedure has been described elsewhere (Castagnola et al., 2013b), but relies on standard optical lithography. The ultra-flexible polyimide micro-ECoG arrays had a total thickness of approximately 8 μm. In spite of their thinness, these devices were mechanically quite robust, making their handling relatively unproblematic. We tested different layouts and electrode sizes (200 × 200 μm and 100 × 100 μm) suitable for different types of neurophysiological experiment. Some designs and details of the electrodes are reported in Figure 12.

**Figure 13:**
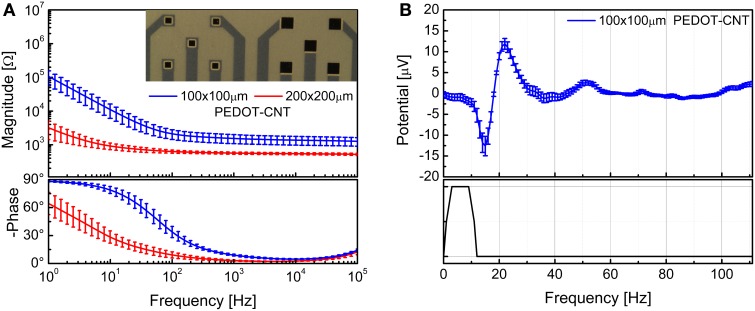
**(A)** Impedance spectra of the 100 × 100 μm and 200 × 200 μm ultraflexible ECoG array recording sites (mean and standard deviation from 10 recording sites each) before and after PEDOT-CNT electrodeposition. **(B)** Averaged SEP elicited using a 9-Hz train of truncated Gaussian 0.8-mm rat multiwhisker deflection (first 12 ms response) using PEDOT-coated 100 × 100 μ m electrodes (average on 10 electrodes).

We were able to selectively coat with PEDOT-CNT HSA composite both 100 × 100 μm and 200 × 200 μm electrodes, demonstrating that the absence of a well in the coverlay does not impair the possibility of obtaining spatially defined coatings, and we obtained reductions in impedance similar to those of our FCP ECoG arrays. Indeed, the impedance at 100 Hz was reduced from 1.6 ± 0.1 MΩ to 2.1 ± 0.7 kΩ with 100 × 100 μm electrodes and from 509.7 ± 177.5 to 0.63 ± 0.07 kΩ with 200 × 200 μm electrodes. The corresponding impedance spectra are shown in Figure 13A. Interestingly, the impedance decreased by up to four orders of magnitude in the 1–100 Hz frequency band (Castagnola et al., 2013b), where ECoG signals are typically recorded, with a significant downshift of the first frequency response pole. In order to validate the recording capability of our ultra-flexible micro-ECoGs, we tested our device *in vivo* by recording from the rat somatosensory cortex, using a stimulation protocol similar to the one described in section Flexible micro-ECoG arrays. An example of averaged SEP elicited using a 9-Hz train of truncated Gaussian 0.8-mm rat multiwhisker deflection (first 12 ms response) using PEDOT-coated 100 × 100 μm electrodes (average on 10 electrodes) is shown in Figure 13B, clearly demonstrating the ability of this device to record clear SEP responses.

## Biocompatibility issues and fear of nanocoatings: a pragmatic approach and proof of concept

The toxicology of nanoparticles is hotly debated in scientific circles, and rather controversial results have been reported. However, experiments often document the *in vitro* or *in vivo* release of dispersed nanoparticles at very high concentrations, making it difficult to assess the real impact of nanomaterial-coated neural probes. Moreover, in the case of carbon nanotubes, a large number of studies report effects that can easily be ascribed to the toxicity of the metal catalyst of unpurified CNTs.

One of the main sources of debate when dealing with nanoparticles, especially CNTs, lies in the fact that, as their properties are largely determined by their surface characteristics, any chemical functionalization will have a very strong influence on the way the particles are metabolized, and therefore any resulting tissue reaction. Some studies have demonstrated that specific types of CNTs can be biodegraded (Kagan et al., 2010; Bianco et al., 2011) even in the brain tissue, after internalization in the microglia (Nunes et al., 2012). The situation becomes even more complex if we consider that in many cases we are dealing with CNT composites, and not isolated particles. If the composite is stable, the surface interacting with the tissue can be considered as a macroscopic particle made of the matrix material, in which the CNTs are fully embedded and encapsulated. If one such composite (macro) particle is released, it may be degraded or not, depending on the material it is made of. If it were undegradable, it would, most probably, be encapsulated as a foreign body. If it were subject to particle degradation, the embedded CNTs would be released into the body in amounts that are vastly smaller than those used in current toxicological experimentation. This makes it very difficult to state whether or not there is a toxic effect at such low concentrations. In the case of nanocoated neural implants, addressing the toxicity of the HSA coating would itself take very long time, and it would be impossible to ascertain with absolute confidence that not one single tiny particle of nanocomposite detaches from the coating during the contact with the brain surface due to mechanical stress. For these reasons we proposed a completely different approach, namely to encapsulate the entire device with a coating that is transparent to ionic currents but, at the same time, protects the device from mechanical stress and prevents, in case of nanocoating failure, the release of particles into the brain tissue. To achieve this we used a hydrogel, specifically human fibrin, to encapsulate our HSA-coated neural micro-ECoG arrays, thereby creating a mechanically stable barrier that prevents direct exposure of the brain to nanomaterials, without, however, impairing their electrochemical and neural performance (Castagnola et al., 2013a,b).

We chose a commercially available two-component fibrin sealant that is approved for surgical purposes in both Europe and the United States (TISSEEL [fibrin sealant], Baxter, USA), and one that is commonly used in neurosurgical practice. We set out to develop a procedure compliant with the protocols approved by the competent medical surveillance agencies for the safe handling and use of fibrin in sterile operating theatres. We devised a process involving the sterilization of the HSA-coated micro-ECoG array before it is introduced to a sterile environment, the fibrin being applied to the device by the surgeon just before use (Castagnola et al., 2013a,b). To deposit the fibrin layer, the polyimide based FPC circuits were placed over an aluminium sample holder heated at 37°C. Meanwhile, the fibrin precursors, fibrinogen, and thrombin, were warmed up to the same temperature. The fibrinogen was spread over the electrodes with a spatula and, then, a large thrombin drop was placed over the whole fibrinogen-coated surface. After 5 min, a stable fibrin layer was formed and the micro–ECoG array was rinsed in ultrapure water to remove the residual thrombin solution.

**Figure 14:**
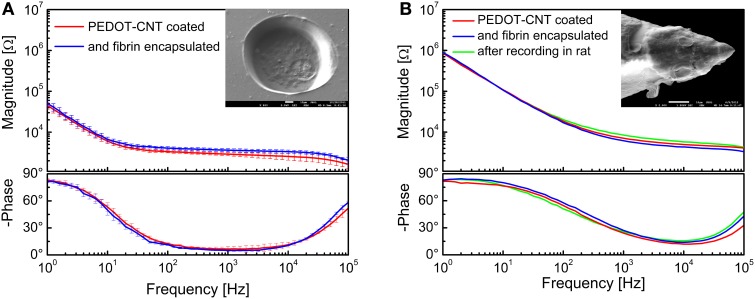
**Impedance spectra of (A) 140-μm-diameter ECoG array recording sites (mean and standard deviation from 64 recording sites each) coated with PEDOT-CNT composite, before and after fibrin encapsulation, and (B) intracortical PEDOT-CNT microelectrode before and after fibrin encapsulation and *in vivo* recording**. s.e.m. images of a single recording/stimulation site encapsulated with fibrin are shown in the respective insets.

A SEM image of a single recording/stimulation site after fibrin encapsulation is shown in Figure 14A, together with the impedance spectra of a 140-μm diameter PEDOT-CNT-coated recording site ECoG array before and after fibrin encapsulation. We can observe that the fibrin encapsulation does not have any significant impact on the impedance spectra of PEDOT-CNT-coated microelectrodes (from 3.4 ± 0.2 kΩ to 3.9 ± 0.4 kΩ @ 100 Hz), confirming that the fibrin hydrogel acts as a good ionic conductor. These fibrin-encapsulated PEDOT-CNT-coated micro-ECoG arrays, being able to resist intense and prolonged current stimulation without the fibrin layer being affected by the current passage (Castagnola et al., 2013a,b) can therefore be considered suitable for cortical stimulation. Indeed, we used these devices *in vivo* to record SEPs from the rat somatosensory cortex, elicited through multiwhisker deflection using a protocol similar to the one reported above. We verified that after two 15-min-long epicortical recording sessions the fibrin coating was still in place, firmly adhering to the recording sites, and thereby effectively shielding the tissues from direct contact with the nanostructured coating. Furthermore, the microelectrode impedance did not change significantly, and the quality of the SEP recordings was not affected by the presence of the fibrin coating (Castagnola et al., 2013a,b).

After proving that the fibrin hydrogel is able to stay in place on the surface of micro-ECoG devices that are placed on the dura mater or cortex, we tested whether or not it can be used to encapsulate an intracortical electrode, and to withstand the mechanical stress generated by brain tissue penetration. To this end, we encapsulated our PEDOT-CNT-coated intracortical microelectrodes by a controlled two-stage dipping process, first into a vial containing fibrinogen, and then into a second vial containing thrombin. The impedance spectra of a PEDOT-CNT-coated electrode before and after fibrin encapsulation, and also after an *in vivo* intracortical recording session involving penetration of the rat brain tissue (including the dura mater), are shown in Figure 14B. A SEM picture of a microelectrode after fibrin encapsulation is shown in the inset. The fibrin encapsulation had no significant effect on the microelectrode impedance, which remained nearly identical, even after the intracortical recording. To check the persistence of the fibrin layer after the rat somatosensory cortex recording session, we stained it with methylene blue, which showed that the fibrin remained adherent to the HSA-coated tip. As previous experiments have shown us that fibrin tends to detach from uncoated, smooth electrodes, this experimental evidence suggests that there is an intimate adhesion between the fibrin layer and the rough HSA coating we applied to the tip (as previously hinted at by electrochemical measurements). In all probability, this adhesion is brought about by the hydrogel being trapped in the nanostructured microelectrode surface, preventing its slipping away.

As previously mentioned, we chose to test fibrin hydrogel due to its potentially ready transferral to human surgery applications. A particular advantage of fibrin hydrogel is that it can be autologous, i.e., obtained from the patient's own blood, thereby reducing to a minimum rejection risks, etc. However, while this approach appears feasible for acute use, unfortunately fibrin can be fully resorbed by the tissues within 2 weeks during *in vivo* implants (Schoeller et al., 2001; de Vries et al., 2002). Nevertheless, our results prove that it is possible to fully encapsulate recording and stimulation devices in hydrogel without impairing their performance, and suggests that it would be possible to extend the use of such a technique to chronic implants by replacing fibrin with an appropriate biocompatible non-resorbable hydrogel with similar physical properties. To this end, it may be advantageous to use synthetic hydrogels as they are better chemically defined, with respect to those composed of only natural materials, and it is possible to better control their polymerization, degradation, and biocompatibility (Aurand et al., 2012a,b). Among all, promising candidates are poly(2- hydroxyethyl methacrylate) (pHEMA) and polyurethane based hydrogels, that have a long-standing track record of successful biomedical applications and have been already tested in contact with the central and pheripheral nervous system (Woerly et al., 1998; Lukás et al., 2001; Dalton et al., 2002; Jhaveri et al., 2009; Rao et al., 2012). To be used for the encapsulation of nanocoated neural chronic implants, it is necessary to accurately fine-tune the hydrogel composition, the cross-linking molecules, and network mesh size, in order to achieve the desired properties in terms of semi-permeability, mechanical stiffness, degree of swelling, and resistance to degradation.

## Perspectives for long term implants

Despite the great efforts and expectations, at present electrodes for brain stimulation and recording are still far from the reliability, affordability, and long term stability necessary for a widespread clinical use in human cortical prosthesis. However, over the last 5 years, by careful selection of readymade technologies, we have been able to modify electrodes to make them smaller, more sensitive, more flexible, less invasive, and more reliable.

With a particular focus on electrodes for human use, we have shown that it is also possible to improve their recording capabilities in terms of sensitivity, signal-to-noise ratio, and electromagnetic interference rejection, by using a variety of HSA coatings based on nanomaterials. We have developed and systematically studied and compared a variety of HSA coatings—CVD-CNT, PPy-CNT, PEDOT-CNT, Au-CNT, Au-agar, PEDOT-agar—proving that such technologies enhance performance in both epicortical and intracortical recording and stimulation. We have also made these coatings mechanically stable and able to withstand penetration through the dura and the brain of an animal model (rat).

Despite the advantages of rigid microprobes, (Campbell et al., 1991; Rousche and Normann, 1992; Richter et al., 2013) particularly in ensuring a precise positioning of the electrodes during insertion into specific brain areas (Schjetnan and Luczak, 2011), they cannot yet provide reliable and stable long-term recordings. Indeed, even state-of-the-art rigid microelectrodes cause severe mechanical trauma during their insertion, resulting in a thick glial scar formation and a consequent loss of tissue-electrode coupling as time goes by (Turner et al., 1999; Polikov et al., 2005; Leach et al., 2010; Richter et al., 2013). That being said, (Krüger et al., 2010) did manage to achieve stable recordings for several years from electrodes experimentally implanted in monkeys thanks to the minimal invasiveness of their device at the cortical recording site. Although this proved the importance of minimizing the mechanical damage to the cortical area under investigation, their electrodes were inserted using a device that enables brain penetration through the white matter from a different area, making the technique very invasive and unsuitable for clinical applications. Hence many other studies have focused on the replacement of rigid with flexible electrodes, which it is hoped will reduce insertion trauma, being more similar mechanically to the soft brain tissue and thereby minimizing the stiffness mismatch between device and nervous system tissue (Rousche et al., 2001; Rubehn and Stieglitz, 2010; Richter et al., 2013). We too aimed to reduce the invasiveness of insertion, by minimizing the physical size of the probe without compromising its electrical impedance, and keeping the tether between the electrode and the external recording/stimulation system as flexible as possible to enable accommodation of brain movement and motions within the skull. We achieved a reduction in probe size by electrochemically growing a HSA gold sphere on the tip of a very thin wire (12 μm in diameter), envisioning the sphere as the ideal shape to be kept in position by the hydrostatic pressure of the brain. This technique also provided us with a very flexible tether, which is not involved in the long term positioning of the device. In a parallel approach, we aimed to overcome the issue of foreign-body response in intracortical implants, developing high-resolution micro-ECoG arrays with enhanced recording capabilities thanks to our HSA coatings. We developed a variety of flexible micro-ECoG arrays of HSA-coated electrodes, whose better signal quality make it feasible to further reduce electrode size and increase spatial resolution. We further improved our technology using novel ultrathin electrodes, which enable intimate contact with the surface of the brain, thereby enhancing the electrical coupling. Finally, we proved that it is possible to reliably encapsulate both intracortical and epicortical devices with a biocompatible hydrogel, which provides a mechanical barrier between the nanocomposite HSA materials, and the human body, without any loss of device performance.

Altogether, these results converge in a method that we believe represents several steps forward in terms of bringing such novel devices into clinical settings, opening new avenues for neurodiagnostics, and neuroprosthetic applications.

### Conflict of interest statement

The authors declare that the research was conducted in the absence of any commercial or financial relationships that could be construed as a potential conflict of interest.

## Footnotes

^1^All animal experimental procedures were approved by the Italian Ministry of Health (Italian Law DL116/92) and were conducted in accordance with the guidelines established by the European Communities Council (Directive 2010/63/EU of September 22nd, 2010).
